# Supplementary material for: SARS-CoV-2 neutralising antibody profiles reveal variant specific antibody dynamics and regional differences in infection histories in Malawi
Source: PLOS Glob Public Health. 2026 Oct 1;6(10):e0007133. doi: 10.1371/journal.pgph.0007133 (PMC13630256; doi:10.1371/journal.pgph.0007133)
Supplement: Supplementary file 1 [file pgph.0007133.s005.pdf]

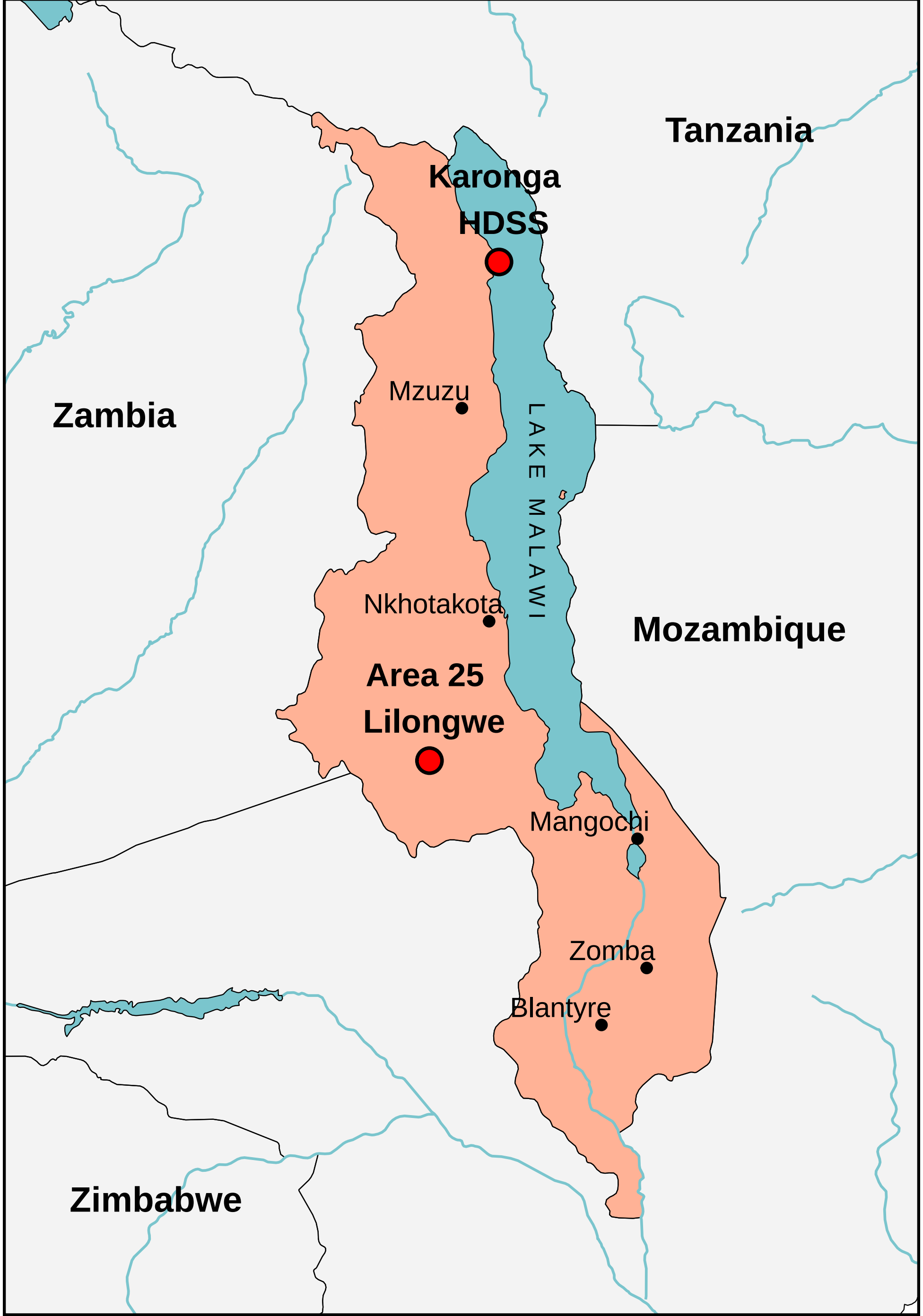
Tanzania
Karonga
HDSS
Zambia
Mzuzu
LAKE MALAWI
Nkhotakota
Mozambique
Area 25
Lilongwe
Mangochi
Zomba
Blantyre
Zimbabwe
