# Supplementary material for: SARS-CoV-2 neutralising antibody profiles reveal variant specific antibody dynamics and regional differences in infection histories in Malawi
Source: PLOS Glob Public Health. 2026 Oct 1;6(10):e0007133. doi: 10.1371/journal.pgph.0007133 (PMC13630256; doi:10.1371/journal.pgph.0007133)
Supplement: Supplementary file 2 [file pgph.0007133.s011.pdf]

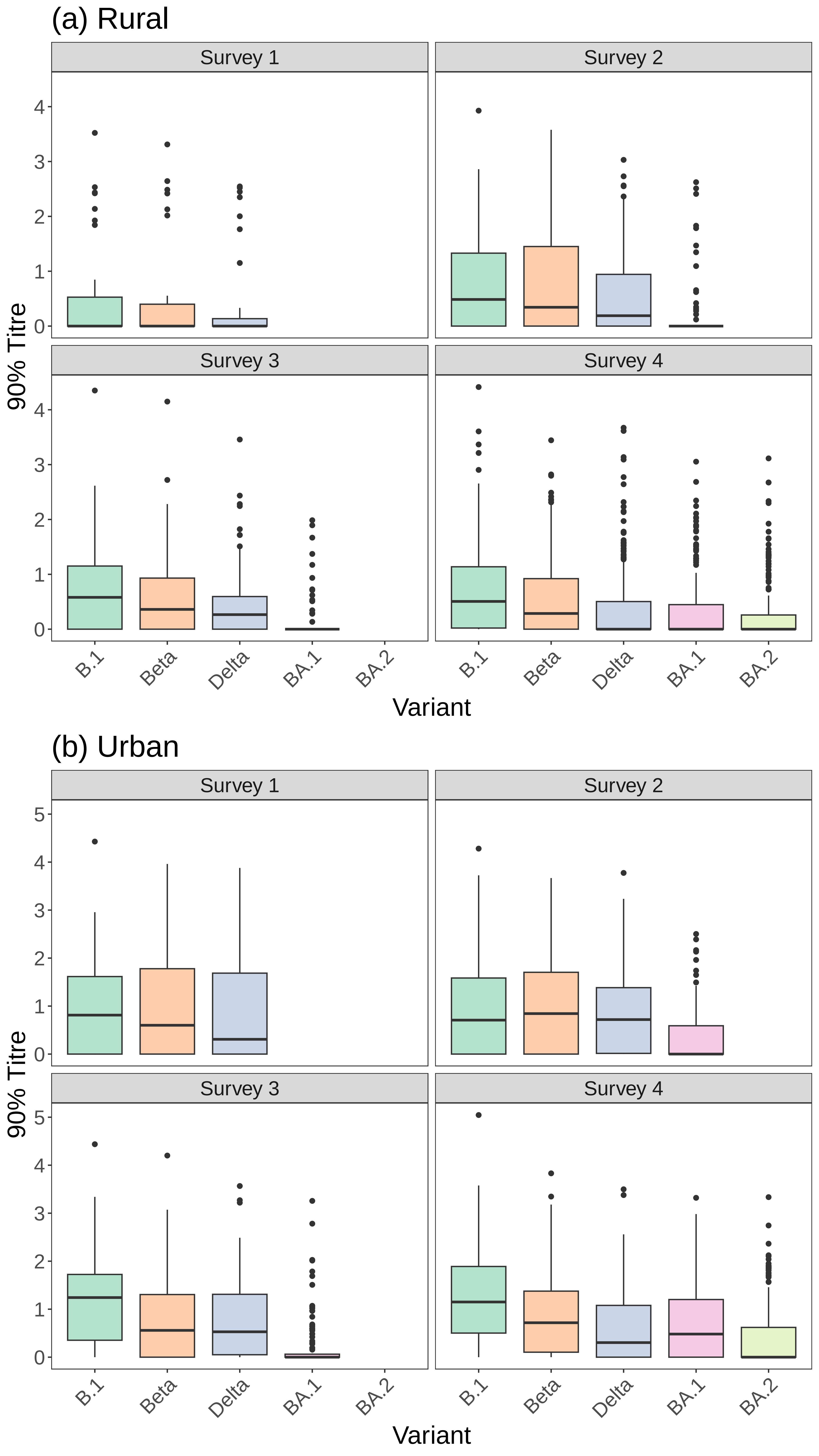

(a) Rural
Survey 1
Survey 2
Survey 3
Survey 4
90% Titre
0
1
2
3
4
B.1
Beta
Delta
BA.1
BA.2
Variant
(b) Urban
Survey 1
Survey 2
Survey 3
Survey 4
90% Titre
0
1
2
3
4
5
B.1
Beta
Delta
BA.1
BA.2
Variant
