# Supplementary material for: SARS-CoV-2 neutralising antibody profiles reveal variant specific antibody dynamics and regional differences in infection histories in Malawi
Source: PLOS Glob Public Health. 2026 Oct 1;6(10):e0007133. doi: 10.1371/journal.pgph.0007133 (PMC13630256; doi:10.1371/journal.pgph.0007133)
Supplement: Supplementary file 3 [file pgph.0007133.s019.pdf]

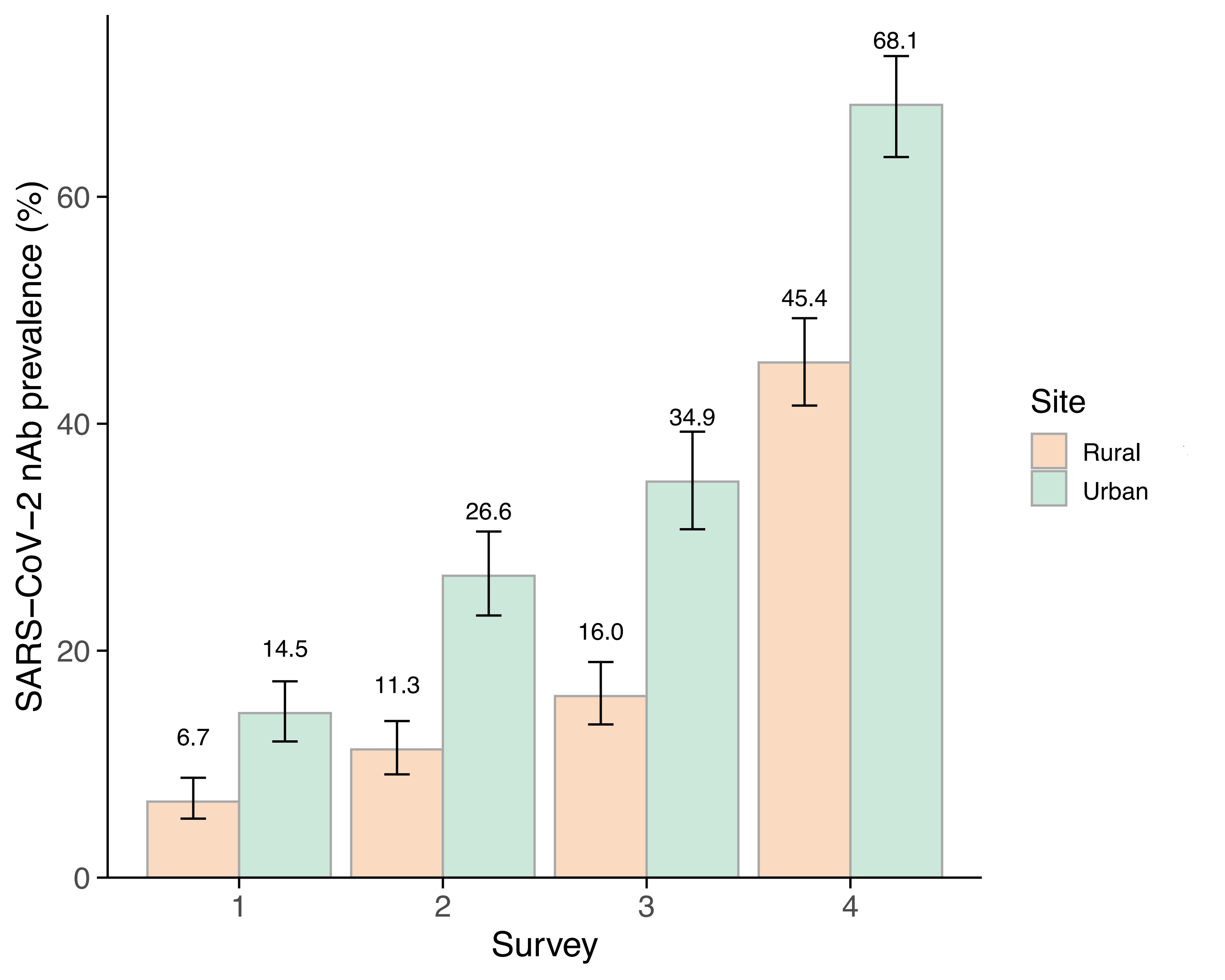
SARS−CoV−2 nAb prevalence (%)
0
20
40
60
6.7
14.5
11.3
26.6
16.0
34.9
45.4
68.1
1
2
3
4
Survey
Site
Rural
Urban
