# Supplementary material for: SARS-CoV-2 neutralising antibody profiles reveal variant specific antibody dynamics and regional differences in infection histories in Malawi
Source: PLOS Glob Public Health. 2026 Oct 1;6(10):e0007133. doi: 10.1371/journal.pgph.0007133 (PMC13630256; doi:10.1371/journal.pgph.0007133)
Supplement: Supplementary file 4 [file pgph.0007133.s020.pdf]

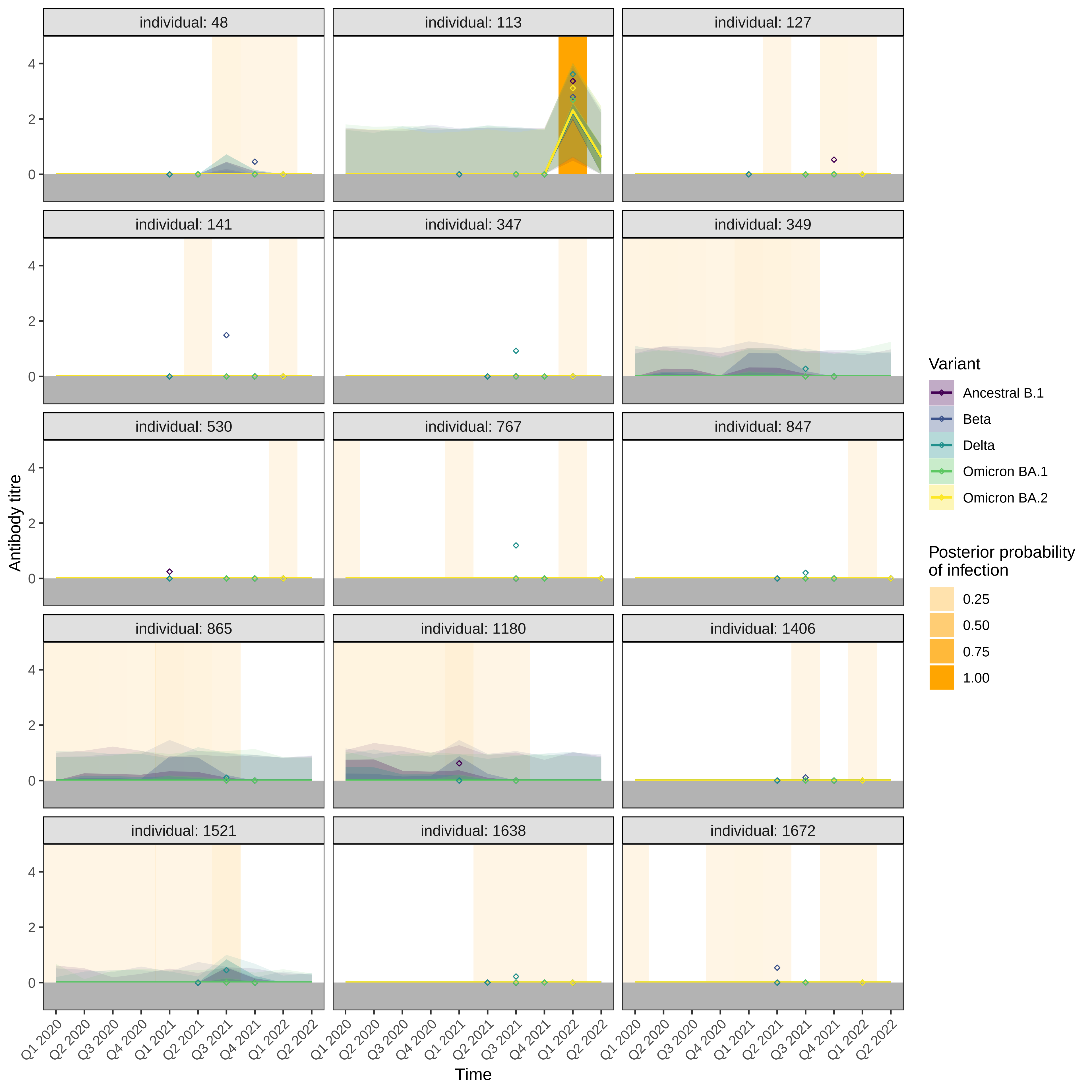
individual: 48
individual: 113
individual: 127
individual: 141
individual: 347
individual: 349
individual: 530
individual: 767
individual: 847
individual: 865
individual: 1180
individual: 1406
individual: 1521
individual: 1638
individual: 1672
Antibody titre
Time
Q1 2020
Q2 2020
Q3 2020
Q4 2020
Q1 2021
Q2 2021
Q3 2021
Q4 2021
Q1 2022
Q2 2022
Variant
Ancestral B.1
Beta
Delta
Omicron BA.1
Omicron BA.2
Posterior probability of infection
0.25
0.50
0.75
1.00
